# Language and cognition in children with metachromatic leukodystrophy: onset and natural course in a nationwide cohort

**DOI:** 10.1186/1750-1172-9-18

**Published:** 2014-02-05

**Authors:** Christiane Kehrer, Samuel Groeschel, Birgit Kustermann-Kuhn, Friederike Bürger, Wolfgang Köhler, Alfried Kohlschütter, Annette Bley, Robert Steinfeld, Volkmar Gieselmann, Ingeborg Krägeloh-Mann

**Affiliations:** 1 Department of Paediatric Neurology and Developmental Medicine, University Children’s Hospital, Hoppe-Seyler-Strasse 1, 72076 Tübingen, Germany; 2 University Children’s Hospital, Metabolic Centre Heidelberg, Metabolic Laboratory, Im Neuenheimer Feld 430, Heidelberg 69120, Germany; 3 Fachkrankenhaus Hubertusburg, Hubertusburg, Wermsdorf 04779, Germany; 4 Department of Paediatrics, University Children’s Hospital Hamburg Eppendorf, Martinistr. 52, Hamburg 20246, Germany; 5 Department of Child and Adolescent Health, Division of Neuropediatrics, University Medical Center Göttingen, Robert-Koch-Str. 40, Göttingen 37075, Germany; 6 Department of Physiology, University of Bonn, Nussallee 11, Bonn 53115, Germany

**Keywords:** Metachromatic leukodystrophy, Natural course, Onset and first symptoms, Decline of language and cognition, Reference data

## Abstract

**Background:**

Metachromatic leukodystrophy (MLD) is a rare, genetic neurodegenerative disease. It leads to progressive demyelination resulting in regression of development and early death. With regard to experimental therapies, knowledge of the natural course of the disease is highly important. We aimed to analyse onset and character of first symptoms in MLD and to provide detailed natural course data concerning language and cognition.

**Methods:**

Patients with MLD were recruited nationwide within the scope of the German research network LEUKONET. 59 patients’ questionnaires (23 late-infantile, 36 juvenile) were analysed.

**Results:**

Time from first symptoms (at a median age of 1.5 years in late-infantile and 6 years in juvenile MLD) to diagnosis took one year in late-infantile and two years in juvenile patients on average. Gait disturbances and abnormal movement patterns were first signs in all patients with late-infantile and in most with juvenile MLD. Onset in the latter was additionally characterized by problems in concentration, behaviour and fine motor function (p = 0.0011, p < 0.0001, and p = 0.0012). Half of late-infantile patients did not learn to speak in complete sentences after an initially normal language acquisition. They showed a rapid language decline with first language difficulties at a median age of 2.5 years and complete loss of expressive language within several months (median age 32, range 22–47 months). This was followed by total loss of communication at a median age of around four years. In juvenile patients, language decline was more protracted, and problems in concentration and behaviour were followed by decline in skills for reading, writing and calculating around four years after disease onset.

**Conclusions:**

Our data reflect the natural course of decline in language and cognition in late-infantile and juvenile MLD in a large cohort over a long observation period. This is especially relevant to juvenile patients where the disease course is protracted and prospective studies are hardly feasible. Knowledge of first symptoms may lead to earlier diagnosis and subsequently to a better outcome following therapeutic intervention. Our data may serve as a reference for individual treatment decisions and for evaluation of clinical outcome after treatment intervention.

## Abstract (de)

**Abstract:**

**Hintergrund**

Die Metachromatische Leukodystrophie (MLD) ist eine seltene, erblich bedingte neurodegenerative Erkrankung. Hauptmerkmal ist eine zentrale und periphere Demyelinisierung, die zum Verlust von Fähigkeiten und vorzeitigem Tode führt. Im Hinblick auf experimentelle Therapieansätze ist die genaue Kenntnis des natürlichen Krankheitsverlaufes unabdingbar. Unser Ziel war es, Zeitpunkt und Symptomatik des Erkrankungsbeginns zu beschreiben, sowie detaillierte Verlaufsdaten des natürlichen Krankheitsverlaufs bezüglich Sprache und Kognition zu etablieren.

**Methodik**

Patienten mit MLD wurden im Rahmen des nationalen Forschungsnetzwerkes LEUKONET rekrutiert. Es wurden die standardisiert erhobenen Fragebogendaten von 59 Patienten mit MLD (23 spätinfantil, 36 juvenil) ausgewertet.

**Ergebnisse**

Die Zeitspanne vom Auftreten erster Krankheitszeichen (im durchschnittlichen Alter von 1,5 Jahren bei spätinfantilen und 6 Jahren bei juvenilen Patienten) bis zur Diagnosestellung betrug ein Jahr bei spätinfantiler und zwei Jahre bei juveniler MLD. Gangstörung und Bewegungsauffälligkeiten kamen zu Beginn der Erkrankung bei allen spätinfantilen und den meisten juvenilen Patienten vor. Bei letzteren kam es typischerweise zusätzlich zu Konzentrationsstörung, Verhaltensauffälligkeiten und feinmotorischen Problemen (p = 0,0011, p < 0,0001, und p = 0,0012). Die Hälfte aller spätinfantilen Patienten lernte nicht, in ganzen Sätzen zu sprechen, obwohl erste Wörter zeitgerecht erlernt worden waren. Es kam zu einem raschen Verfall der Sprache mit ersten Auffälligkeiten um das mittlere Alter von 2,5 Jahren bis hin zum völligen Verlust der gesprochenen Sprache binnen weniger Monate (Median 32, Range 22–47 Monate). Mit ca. vier Jahren zeigte sich ein Verlust jeglicher Kommunikation. Bei juvenilen Patienten begann der Sprachabbau später und verlief verzögert. Probleme in Konzentration und Verhalten waren gefolgt von Schwierigkeiten im Lesen, Schreiben und Rechnen ca. vier Jahre nach Erkrankungsbeginn.

**Konklusion**

Unsere Daten spiegeln den natürlichen Krankheitsverlauf bei spätinfantiler und juveniler MLD im Hinblick auf Verlust von Sprache und Kognition in einer großen Patientenkohorte über einen längeren Beobachtungszeitraum wider. Dies ist insbesondere für juvenile Patienten bedeutsam, deren Erkrankung so langsam verläuft, dass Langzeitstudien kaum durchführbar sind. Die genaue Kenntnis erster Krankheitszeichen kann eine frühe Diagnose ermöglichen und so den klinischen Nutzen nach experimenteller Therapie verbessern. Unsere Daten können als Referenzdaten in der Beurteilung individueller Therapieempfehlung oder in der Evaluation nach therapeutischer Intervention dienen, wo es bislang kaum verbindliche Kriterien gibt.

## Background

Metachromatic leukodystrophy (MLD) is a rare, inherited neurodegenerative disease caused by deficiency of arylsulfatase A (ASA), or - more rarely - of its activator protein saposin-B. Progressive demyelination in the central and peripheral nervous system results in developmental stagnation or regression and in various neurological symptoms [1-3].

With respect to the age of onset, a late-infantile, a juvenile and an adult form of the disease are distinguished. Clinical manifestation in the late-infantile form is characterized by hypotonic or hypertonic paresis starting in the legs and regression of motor and mental function. Prognosis is severe, leading to death within a few years [3]. In the juvenile form, difficulties in school may precede these symptoms [2].

There is currently no specific treatment for MLD. Stem cell transplantation (SCT), when performed at an early stage of the disease, may improve the outcome of patients with a juvenile form of MLD [3-6]. Furthermore, new therapeutic options such as enzyme replacement (ER) and gene therapy are topics of current research [7-9]. Especially with regard to new therapies and the need for long-term follow-up-studies, detailed knowledge of the natural course of the disease is of high interest. Deterioration of gross motor function is a key feature of the disease, and we have analysed the natural course of gross motor deterioration in late-infantile and juvenile MLD in a previous study [10,11]. Cognitive decline accompanies motor deterioration and may precede motor signs in patients with later onset. In the present study, we therefore aimed to analyse onset and character of first symptoms of the disease as well as to provide natural course data concerning expressive language and cognition in late-infantile and juvenile MLD.

## Methods

### Study group

Patients were recruited nationwide within the scope of the German research network LEUKONET during a period of 5 years by contacting patients’ organizations and all German laboratories performing ASA determination, as previously described [11].

Standardized patients’ questionnaires were analysed from a total of 59 untreated, affected patients (32 male, 27 female), 23 with a late-infantile, 36 with a juvenile form of MLD. Onset was defined as first neurological symptoms and/or decline of motor, cognitive or behavioural function. Age of onset was defined as younger than 30 months for late-infantile and from 30 months to below 16 years for juvenile cases [12].

MLD was diagnosed in all patients based on ASA-deficiency as well as typical leukodystrophic signs in initial MRI [13,14] together with typical clinical symptoms. In addition, investigation of the urinary sulfatide level was done in 44 patients showing increased sulfatide levels in all of those 44 cases, and molecular analysis of DNA was done in 31 patients showing two pathogenic mutations in all of those 31 cases.

Informed consent was given by the parents in all cases. The study was approved by the Ethical Committee of the University of Tübingen (Nr. 401/2005).

### Data source

Data on natural course of MLD were collected prospectively and retrospectively. The data source consisted of hospital records and standardized patients’ questionnaires. Furthermore, the parents of patients as well as local physicians were periodically interviewed over the telephone by the same investigator (C.K.). Data of six patients, who had already deceased prior to the beginning of the study, were collected exclusively retrospectively. All data were double checked and monitored by a third party (Center for Pediatric Clinical Studies (CPCS), Tübingen, Germany).

### Analysis of language and cognition

In order to describe *acquisition of language abilities*, we analysed the age of acquisition of a) single meaningful words, b) two-word-sentences, c) complete sentences. In order to describe *regression of language abilities*, we analysed age at: a) loss of complete sentences, b) loss of two-word-sentences, c) loss of single meaningful words, d) first language decline, e) complete loss of expressive language. In order to describe *regression of cognitive abilities*, we analysed the age when the child presented with a) problems in concentration, b) behavioural problems, c) decline in skills for reading, writing and calculating, d) loss of any communication. Parents were instructed to assess item a) “problems in concentration”, if there were any aspects of an attention disorder covering poor concentration, lack of endurance, diminished attention or slower working speed. Parents were instructed to assess item b) “behaviour problems”, if they noted bad temper, moods, unsocial or aggressive behaviour. Item c) had to be assessed, if there were problems concerning at least one of the three parameters “reading”, “writing” or “calculating”. Parents were instructed to assess item d) “loss of any communication”, if they noted complete loss of verbal communication combined with loss of directed voluntary movements, reaction towards optic/acoustic stimuli as well as loss of the ability to fix and follow with the eyes.

### Statistics

Data are summarized with the median and inter-percentile ranges (25th and 75th percentiles). To compare categorical or binary variables across the forms of MLD, the Cochrane-Mantel-Haentzel-Test was employed. Time until loss of any function was estimated by time-to-event analysis using the Kaplan-Meier method. Comparisons between MLD forms were based on the Log Rank Test and the Wilcoxon Test.

## Results

### Onset and diagnosis

Medians (and ranges) of age of onset were 17 (9–27) months for the late-infantile group (n = 23), and 76 (32–162) months for the juvenile group (n = 36). The median time between onset (first symptoms) and diagnosis was 12 months (range 2–21) in the late-infantile form and 21 months (range 1–282) in the juvenile form (p < 0.01).

First symptoms of the 23 late-infantile and 36 juvenile patients are shown in Table 1. “Gait disturbances” and “abnormal movement patterns” were the most frequent first symptoms in both forms of MLD. None of the late-infantile, but six juvenile patients (17%) had exclusively “non gross motor” symptoms as first signs of the disease. There were no differences in the frequency of gait disturbance, weakness and abnormal movement patterns (also pain and irritability) as first signs between the two forms. On the other hand, “impaired fine motor skills”, “concentration problems”, and “behavioural problems” as first symptoms occurred more often in juvenile MLD (p = 0.0011, p < 0.0001, and p = 0.0012, respectively). These three symptoms in combination occurred in 36% of the juvenile patients.

**Table 1:** First symptoms of the disease in late-infantile and juvenile MLD

**First symptoms**	**Late-infantile (n = 23)**		**Juvenile (n = 36)**		**p**
	**n**	**%**	**n**	**%**	
Gait disturbance	16	70%	25	69%	0.9922
Pain	6	26%	5	14%	0.2447
Abnormal movement patterns	14	61%	25	69%	0.5011
Impaired fine motor skills	4	17%	22	61%	0.0011
Restlessness/irritability	4	17%	10	28%	0.3645
Weakness	10	43%	13	36%	0.5748
Problems in concentration	0	0%	23	64%	0.0001
Behavioural problems	3	13%	20	56%	0.0012
Developmental regression in general	14	61%	19	53%	0.5449

### Language acquisition

All juvenile patients learned to speak *complete sentences.* All late-infantile patients acquired *single meaningful words.* 4 late-infantile patients (17%) never acquired speaking in *two-words-sentences* and 11 late-infantile patients (48%) never learned to speak in *complete sentences*. For those who reached a certain ability, language acquisition was within the normal age range for healthy term born children [15].

### Regression of language and cognitive abilities

**Figure 1:**
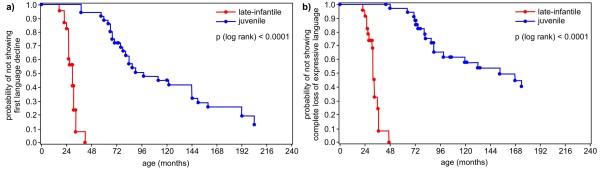
**Kaplan–Meier plots show age (months) when a) *first language decline *** **and b) *complete loss of expressive language *** **occurred in patients with the late-infantile form of metachromatic leukodystrophy compared with those with the juvenile form (degrees of freedom = 1).**

**Figure 2:**
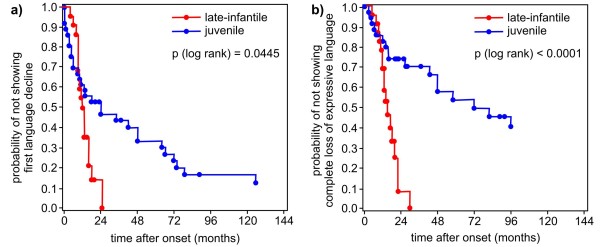
**Kaplan–Meier plots show time after onset (months) when a) *first language decline *** **and b) *complete loss of expressive language *** **occurred in patients with the late-infantile form of metachromatic leukodystrophy compared with those with the juvenile form (degrees of freedom = 1).**

**Figure 3:**
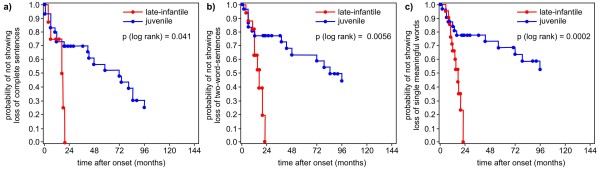
**Kaplan–Meier plots show time after onset (months) when a) *loss of complete sentences, *** **b) *loss of two-word-sentences, *** **and c) *loss of single meaningful words *** **occurred in patients with the late-infantile form of metachromatic leukodystrophy compared with those with the juvenile form (degrees of freedom = 1).**

**Figure 4:**
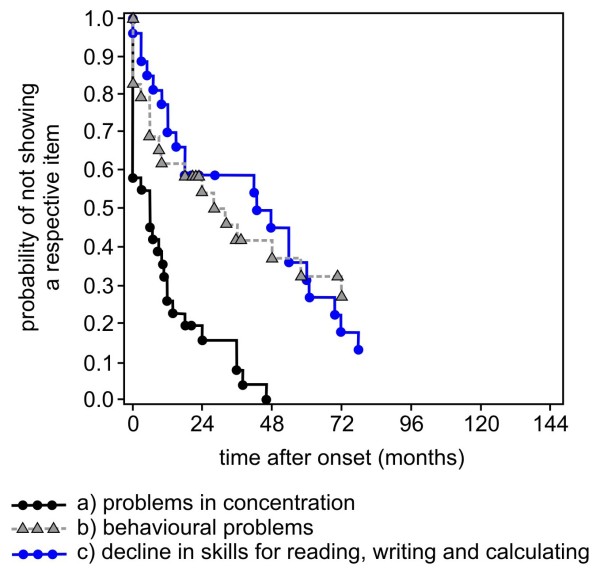
**Kaplan–Meier plots show time after onset (months) when *regression of cognitive abilities *** **in patients with the juvenile form of MLD occurred with respect to the items a) problems in concentration, b) behavioural problems, and c) decline in skills for reading, writing and calculating.**

**Figure 5:**
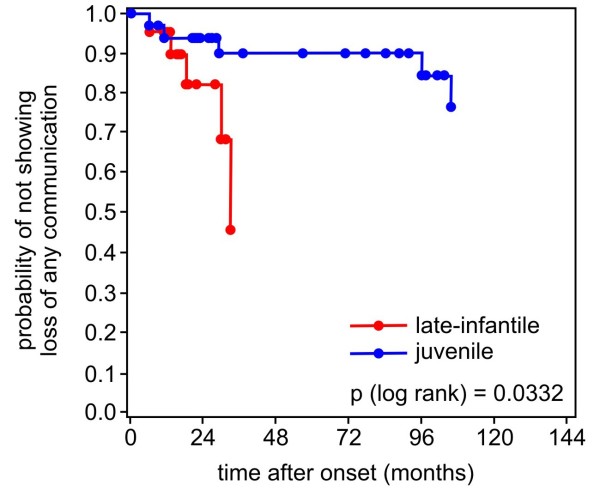
**Kaplan–Meier plots show time after onset (months) when *loss of any communication *** **occurred in patients with the late-infantile form of metachromatic leukodystrophy compared with those with the juvenile form (degrees of freedom = 1).**

Age and time after onset (median and 25th/75th percentile) of parameters of cognitive and language decline are shown in Table 2 for both forms of MLD. Kaplan-Meier estimates show the age and the time after onset, when a certain ability is lost concerning language and cognition in the late-infantile compared to the juvenile form (Figure 1, 2, 3, 4 and 5). Decline of language and cognition started later and much more variable in the juvenile form not only when considering age but also with respect to disease onset. Language decline occurred in the late-infantile form at a median age of 30 months (range 17–42 months), and complete loss of language occurred at a median age of 32 months (range 22–47 months). This corresponds to a median time after onset of 12 months (language decline) and 17 months (complete loss of language). In the juvenile form, language decline and complete loss of language were observed at a median age of 8 and 13 years respectively, and at a median time after onset of 2 and 6 years respectively. Decline in cognition began in the juvenile form at a median age of 7 years with problems in concentration; decline in skills for reading, writing and calculating then occurred at a median age of 10.5 years, four years after onset. While the ability of any communication was lost at the age of just over four years in the late-infantile form, this was observed at the age of 16 years in the juvenile form (25th percentile, median not reached).

**Table 2:** Age and time after onset of language and cognitive decline in late-infantile and juvenile MLD

**a) Age [Months]**	**Late-infantile**			**Juvenile**			**p**
	**P25**	**Median**	**P75**	**P25**	**Median**	**P75**	
Loss of complete sentences	28	30	31	84	132	- - -	<0,0001
Loss of two-word-sentences	30	32	36	90	174	- - -	<0,0001
Loss of single meaningful words	31	32	36	98	- - -	- - -	<0,0001
First language decline	26	30	31	69	98	192	<0,0001
Complete loss of expressive language	28	32	36	87	153	- - -	<0,0001
Problems in concentration	31	- - -	- - -	62	84	113	0,0808
Behavioural problems	28	31	- - -	78	142	192	<0,0001
Decline in reading, writing and calculating	- - -	- - -	- - -	87	128	180	- - -
Loss of any communication	48	51	- - -	192	- - -	- - -	0,0011
**b) Time after onset [Months]**	**Late-infantile**			**Juvenile**			**p**
	**P25**	**Median**	**P75**	**P25**	**Median**	**P75**	
Loss of complete sentences	11	17	19	12	72	- - -	0,0409
Loss of two-word-sentences	12	17	20	38	96	- - -	0,0056
Loss of single meaningful words	11	17	20	43	- - -	- - -	0,0002
First language decline	9	12	16	5	24	72	0,0445
Complete loss of expressive language	12	15	20	16	72	- - -	<0,0001
Problems in concentration	16	- - -	- - -	0	6	14	<0,0001
Behavioural problems	9	18	21	6	32	- - -	0,4415
Decline in reading, writing and calculating	- - -	- - -	- - -	12	48	70	- - -
Loss of any communication	30	33	- - -	- - -	- - -	- - -	0,0332

Parameters of regression of language and cognitive abilities correspond to the parameters introduced in the Methods section. P25 indicates the 25th percentile, median indicates the 50th percentile and P75 indicates the 75th percentile. Age and time after onset are given in months.

## Discussion

To our knowledge, this study comprises the largest cohort of patients with MLD worldwide and is the first to give a standardized description of parameters characterizing language, cognition and behaviour during disease course. Data were obtained nationwide within the scope of the German Leukonet.

### Onset and diagnosis

While it took around one year from first symptoms to diagnosis in late-infantile MLD, this was nearly doubled in juvenile patients. Possibly, first symptoms in juvenile MLD are not quickly recognized as disease indicators, although only the minority (17%) start with rather unspecific signs such as behavioural and concentration problems (see below). Time from onset to diagnosis has been reported to be even longer in a retrospective study on 22 late-infantile and 4 juvenile Brazilian patients with MLD [16], where it took a median time of 20 months after onset in late-infantile MLD and over six years in the juvenile group. Considering that patients with a juvenile form seem to benefit from therapeutic intervention like SCT, when performed early in the disease course [4,6,17], it is of great clinical importance to draw attention to the typical first signs to shorten the long time period between first symptoms and diagnosis particularly in this group. Early diagnosis will enable early individual treatment and hopefully lead to a better outcome.

Gait disturbances and abnormal movement patterns were the leading first symptoms in both groups of MLD in our cohort. Although late-onset MLD is described to start with school problems or psychiatric symptoms [2,18], it seems noteworthy that most of juvenile patients in our cohort also presented with motor symptoms as first signs of the disease, which seems an important observation when it concerns efforts to accelerate early diagnosis. Only six of them presented exclusively “non gross motor” signs as first symptoms. Although less frequent as first signs, prevalence of weakness, pain and irritability did not differ between our two groups. In contrast, problems in concentration and behaviour, as well as impaired fine motor skills occurred significantly more often as first symptoms in the juvenile form. The combination of these three symptoms (partially in addition to gross motor symptoms) was present in 36% of juvenile patients, which seems to be a characteristic feature of onset in juvenile MLD.

### Language acquisition

Moser described a normal early language acquisition in late-infantile MLD [12]. In our study-group, patients of both forms of MLD learned single words and most of them also two-word-sentences without delay. However, about half of the late-infantile patients never learned to speak in complete sentences after having acquired one- and two-word-sentences within the normal time range. We believe this is a phenomenon not recognized so far, and absence of acquisition of complete sentences after initial normal language acquisition may be a characteristic finding in late-infantile MLD. Therefore, such a finding should lead to further investigation. The observed stagnation in language development around 2.5 years of age corresponds to the time period where decline of gross motor function in patients with late-infantile MLD takes place [11]. Language acquisition was completely normal in all patients with juvenile MLD; thus it is noteworthy, underlined by our report on normal motor milestones [11], that patients, who develop MLD after the age of 30 months, show a normal development before onset of their disease.

### Language regression

In the late-infantile form, first language difficulties occurred at a median age of about 2.5 years, around 1 year after onset (Figures 1a, 2a and 3a). Complete loss of speech occurred before the age of 3 years on average. All late-infantile patients had completely lost their expressive abilities before the age of 4 years, between 2 and 2.5 years after onset, and none were able to speak any single word (Figures 1b, 2b and 3b-c). It was a new and striking finding, that – on average - loss of language after first language difficulties took place within several months. Thus, the progression of language decline was even faster than gross motor decline, where regression from first gross motor problems to loss of all gross motor function within a median of 15 months is reported [11]. Our data support an observation of Hagberg [19], who related loss of language in late-infantile MLD to his so-called stage III, where “speech has disappeared more or less completely” and “tetraplegia, absent tendon reflexes and increasing mental retardation” characterized the clinical picture, occurring after about 1.5 years of onset. Moser [12] reported a “regression of language” within 6 months to 2.5 years after onset in late-infantile patients, but this was not described in detail. Biffi et al. [8] recently described normal language in three late-infantile patients after lentiviral HSC gene therapy between the age of 25 to 39 months, where their affected siblings were “incapable of any voluntary speech”. In our natural course study the age range, where language decline started, reached up to 42 months in the late-infantile form (see Figure 1a), indicating that their treated patients are not yet beyond the here shown age range for first signs of language decline. This illustrates that reference data of the natural course of the disease - which allow the comparison not only to an affected sibling of any index patients but also to a whole patient cohort - are extremely important in the evaluation of clinical outcome in new therapies and long-time observation.

Juvenile patients, on average six years of age at the onset of their disease, declined later in language abilities than late infantile patients: First language difficulties occurred with a median time after onset of two years in this group and complete loss of expressive language after six years. Both parameters were much more variable in the juvenile group than in the late-infantile group not only with respect to age, but also to time after onset (Figures 2 and 3).

### Cognition

Over 60% of patients with juvenile MLD presented with difficulties in concentration at onset of their disease, which indicates that these are typical first symptoms in this group. The items reported by parents were predominantly diminished attention and lack of endurance, but in school children, poor concentration in general as well as slower working speed were also reported. When other symptoms preceded, problems in concentration followed within one year in 75% of these patients (Figure 4).

In addition, behavioural problems were reported by the parents at an early stage in both forms of MLD and occurred within the first two years after disease onset. Although not formally analysed, parents’ responses suggested that behavioural problems in the late-infantile form means that children were moody and ill-tempered, while in the juvenile form antisocial or aggressive behaviour was predominant. In the juvenile form of MLD, behavioural symptoms were among the most frequent first symptoms.

Decline in skills for reading, writing and calculating, was not relevant in late-infantile MLD and also in 6 juvenile patients as disease started too early. However, it was reported in about 80% of the remaining juvenile patients. Problems in reading, writing and calculating followed the more unspecific symptoms like problems in concentration and behaviour, and occurred at a median age of ten years, about four years after onset (Figure 3). These typical problems in school represent the cognitive decline in juvenile patients and usually do not remain undiscovered. Decline in skills for reading, writing and calculating often led to changes in school career such as repeating a year, changing of school form or even leaving school, but data of school forms unfortunately often were insufficient and could not be obtained systematically in this cohort.

Learning disabilities and decline in school performance are known to be typical symptoms in the juvenile form of the disease [2,3,20]. Behavioural disorders are said to characterize disease onset in the late onset forms [18]. Clarke et al. described the mental decline in a girl with behavioural problems at first symptoms at the age of 9 years and a following decline of IQ from 110 to 87 and to 52 within 3 and 5 years [21]. In our cohort, there were also juvenile patients, in whom mental decline was documented in detail by standardized neuropsychological tests (HAWIVA III, HAWIK IV, K-ABC, SON-R 2,5-7, WPPSI III) from initial normal IQ to mild and severe mental disability. But due to the retrospective analysis of developmental data, time-point and testing were not standardized, so that standardized and quantified evaluation of mental decline in a representative cohort was not possible. To our knowledge, there are no systematically assessed data of impairment in neuropsychological testing in patients with MLD in the literature so far.

Loss of any communication was observed in half of the late-infantile patients at the age of just over four years (- until three years after onset -) and in a quarter of the juvenile patients until nine years after onset (Figure 5). It seems remarkable that parents tended to judge their children as capable of some form of interaction even when they were completely bed-ridden, blind, and had no expressive language.

## Conclusions

Gait disturbances and abnormal movement pattern were predominant as first signs in both forms of MLD. While motor problems alone are typical for the onset of the late-infantile form, the juvenile form is characterized in addition by problems in concentration, behaviour and fine motor function. Patients with a juvenile onset showed completely normal development of language milestones, whereas half of late-infantile patients did not learn to speak in complete sentences after an initially normal language acquisition. Language decline was rapid in late-infantile patients with first language difficulties at a median age of about 2.5 years to complete loss of expressive language within several months. This was followed by loss of any communication at a median age of around 4 years. In juvenile patients, language decline was more variable and showed a more protracted course. The first and unspecific symptoms of concentration and behavioural problems were followed by decline in skills for reading, writing and calculating around four years after onset in this group.

Our data reflect the natural course of language and cognition in late-infantile and juvenile MLD in a large cohort over a long observation period. We chose the approach to rely on mainly retrospective data acquired by the same investigator in a standardized setting. This allowed an overview on disease courses over a long period of time, in many cases including end-point-observations. This seems especially relevant for juvenile cases, where the disease course can be very protracted and prospective studies are hardly feasible. Our data may help to raise awareness of early symptoms and may lead to a better knowledge of the clinical picture of early disease stages what, consequently, might result in earlier diagnosis. Increasing numbers of patients with MLD undergo SCT worldwide, but well-established in- and exclusion criteria and mandatory evaluation standards are lacking. Knowledge of the natural history seems essential for individual treatment decisions, as it allows to judge how advanced the patient’s disease stage is. Our natural-course-data will allow the comparison between the clinical outcome after intervention and the clinical features of the untreated cohort and may therefore allow judgement of therapeutic effects. Thus, our data may serve as a reference not only for individual treatment decisions but also for evaluation of therapies.

## Abbreviations

ASA: Arylsulfatase A; ER: Enzyme replacement; HAWIK: Hamburg-Wechsler-Intelligenztest für Kinder; HAWIVA: Hannover-Wechsler-Intelligenztest für das Vorschulalter; IQ: Intelligence quotient; K-ABC: Kaufman assessment battery for children; MLD: Metachromatic leukodystrophy; MRI: Magnet resonance imaging; SCT: Stem cell transplantation; SON-R 2,5-7: Snijders-Oomen Nicht-verbaler Intelligenztest; WPPSI III: Wechsler preschool and primary scale of intelligence - third edition.

## Competing interests

The authors declare that they have no competing interests.

## Authors’ contributions

CK made substantial contributions to conception and design of the study as well as to acquisition and interpretation of data and was involved in recruitment of patients. She studied the medical records, was responsible for standardised interviews by phone and drafted the paper. SG helped with the statistical analysis and interpretation of data and revised the manuscript critically for important intellectual content. BK and FB carried out biochemical analysis of measurement of Arylsulfatase A in leucocytes and sulfatides in urine and were substantially involved in recruitment of patients. WK was responsible for recruitment of patients with a juvenile form of MLD and acquisition and interpretation of data of natural course in juvenile MLD. AK was involved in the design of a standardised patients’ questionnaire. AB and RS were involved in recruitment of patients and acquisition of data. VG participated in the design of the study and the manuscript. IK-M was involved in conception and design of the study as well as in drafting the manuscript and gave final approval of the version to be published. All authors read and approved the final manuscript.

## References

[B1] Gieselmann V Metachromatic leukodystrophy: genetics, pathogenesis and therapeutic options Acta Paediatr Suppl 2008 97 15 21 10.1111/j.1651-2227.2008.00648.x 18339182

[B2] von Figura K Gieselmann V Jaeken J Scriver CR, Beaudet AL, Sly WS, Valle D Metachromatic leukodystrophy The Metabolic And Molecular Bases Of Inherited Disease. Chapter 148 2001 New York: McGraw-Hill 3695 3724

[B3] Gieselmann V Krägeloh-Mann I Metachromatic leukodystrophy - an update Neuropediatrics 2010 41 1 6 10.1055/s-0030-1253412 20571983

[B4] Martin HR Poe MD Provenzale JM Kurtzberg J Mendizabal A Escolar ML Neurodevelopmental outcomes of umbilical cord blood transplantation in metachromatic leukodystrophy Biol Blood Marrow Transplant 2013 19 616 624 10.1016/j.bbmt.2013.01.010 23348427

[B5] van Egmond ME Pouwels PJ Boelens JJ Lindemans CA Barkhof F Steenwijk MD van Hasselt PM van der Knaap MS Wolf NI Improvement of white matter changes on neuroimaging modalities after stem cell transplant in metachromatic leukodystrophy JAMA Neurol 2013 70 779 782 10.1001/jamaneurol.2013.629 23608771

[B6] Krägeloh-Mann I Groeschel S Kehrer C Opherk K Nägele T Handgretinger R Müller I Juvenile metachromatic leukodystrophy 10 years post transplant compared with a non-transplanted cohort Bone Marrow Transplant 2013 48 369 375 10.1038/bmt.2012.155 22941383

[B7] Biffi A Aubourg P Cartier N Gene therapy for leukodystrophies Hum Mol Genet 2011 20 R42 53 10.1093/hmg/ddr142 21459776

[B8] Biffi A Montini E Lorioli L Cesani M Fumagalli F Plati T Baldoli C Martino S Calabria A Canale S Benedicenti F Vallanti G Biasco L Leo S Kabbara N Zanetti G Rizzo WB Mehta NA Cicalese MP Casiraghi M Boelens JJ Del Carro U Dow DJ Schmidt M Assanelli A Neduva V Di Serio C Stupka E Gardner J von Kalle C Bordignon C Ciceri F Rovelli A Roncarolo MG Aiuti A Sessa M Naldini L Lentiviral hematopoietic stem cell gene therapy benefits metachromatic leukodystrophy Science 2013 341 1233158 10.1126/science.1233158 23845948

[B9] Batzios SP Zafeiriou DI Developing treatment options for metachromatic leukodystrophy Mol Genet Metab 2012 105 56 63 10.1016/j.ymgme.2011.10.002 22078456

[B10] Kehrer C Blumenstock G Raabe C Krägeloh-Mann I Development and reliability of a classification system for gross motor function in children with metachromatic leucodystrophy Dev Med Child Neurol 2011 53 156 160 10.1111/j.1469-8749.2010.03821.x 21087233

[B11] Kehrer C Blumenstock G Gieselmann V Krägeloh-Mann I German L The natural course of gross motor deterioration in metachromatic leukodystrophy Dev Med Child Neurol 2011 53 850 855 10.1111/j.1469-8749.2011.04028.x 21707604

[B12] Moser H Lees M Stanbury JB, Wyngaarden JB, Fredrickson DS Sulfatide lipidosis: metachromatic leukodystrophy The metabolic basis of inherited disease 1965 New York: McGraw-Hill 539 559

[B13] Groeschel S Kehrer C Engel C Dali CI Bley A Steinfeld R Grodd W Krägeloh-Mann I Metachromatic leukodystrophy: natural course of cerebral MRI changes in relation to clinical course J Inherit Metab Dis 2011 34 1095 1102 10.1007/s10545-011-9361-1 21698385

[B14] Groeschel S Dali CI Clas P Böhringer J Duno M Krarup C Kehrer C Wilke M Krägeloh-Mann I Cerebral gray and white matter changes and clinical course in metachromatic leukodystrophy Neurology 2012 79 1662 1670 10.1212/WNL.0b013e31826e9ad2 22993277 PMC4098858

[B15] Largo RH Molinari L Comenale Pinto L Weber M Duc G Language development of term and preterm children during the first five years of life Dev Med Child Neurol 1986 28 333 350 3721077 10.1111/j.1469-8749.1986.tb03882.x

[B16] Artigalás O Lagranha VL Saraiva-Pereira ML Burin MG Lourenço CM van der Linden H Jr Santos ML Rosemberg S Steiner CE Kok F de Souza CF Jardim LB Giugliani R Schwartz IV Clinical and biochemical study of 29 Brazilian patients with metachromatic leukodystrophy J Inherit Metab Dis 2010 33 Suppl 3 S257 62 20596894 10.1007/s10545-010-9140-4

[B17] Görg M Wilck W Granitzny B Suerken A Lukacs Z Ding X Schulte-Markwort M Kohlschütter A Stabilization of juvenile metachromatic leukodystrophy after bone marrow transplantation: a 13-year follow-up J Child Neurol 2007 22 1139 1142 10.1177/0883073807306256 17890417

[B18] Shapiro EG Lockman LA Knopman D Krivit W Characteristics of the dementia in late-onset metachromatic leukodystrophy Neurology 1994 44 662 665 10.1212/WNL.44.4.662 8164821

[B19] Hagberg B Folch-Pi J, Bauer H Clinical symptoms, signs and tests in metachromatic leucodystrophy Brain Lipids And Lipoproteins, And The Leucodystrophies 1963 Amsterdam - London - New York: Elsevier 134 146

[B20] Lyon G Kolodny EH Pastores GM Neurology Of Hereditary Metabolic Diseases Of Children 2006 3 New York - Chicago - San Francisco: McGraw-Hill

[B21] Clarke JT Skomorowski MA Chang PL Marked clinical difference between two sibs affected with juvenile metachromatic leukodystrophy Am J Med Genet 1989 33 10 13 10.1002/ajmg.1320330104 2568751

